# A preliminary proteomic characterisation of extracellular vesicles released by the ovine parasitic nematode, *Teladorsagia circumcincta*

**DOI:** 10.1016/j.vetpar.2016.03.008

**Published:** 2016-05-15

**Authors:** Thomas Tzelos, Jacqueline B. Matthews, Amy H. Buck, Fabio Simbari, David Frew, Neil F. Inglis, Kevin McLean, Alasdair J. Nisbet, C. Bruce A. Whitelaw, David P. Knox, Tom N. McNeilly

**Affiliations:** a Moredun Research Institute, Pentlands Science Park, Bush Loan, EH26 0PZ, Edinburgh, UK; b Institute of Immunology and Infection Research, School of Biological Sciences, University of Edinburgh, King’s Buildings, EH9 3JL, Edinburgh, UK; c The Roslin Institute, University of Edinburgh, Easter Bush, Midlothian,EH25 9RG, Edinburgh, UK

**Keywords:** Nematode, *Teladorsagia circumcincta*, Proteomics, Extracellular vesicles

## Abstract

*Teladorsagia circumcincta* is a major cause of ovine parasitic gastroenteritis in temperate climatic regions. The development of high levels of anthelmintic resistance in this nematode species challenges its future control. Recent research indicates that many parasite species release extracellular vesicles into their environment, many of which have been classified as endocytic in origin, termed exosomes. These vesicles are considered to play important roles in the intercellular communication between parasites and their hosts, and thus represent potentially useful targets for novel control strategies. Here, we demonstrate that exosome-like extracellular vesicles can be isolated from excretory-secretory (ES) products released by *T. circumcincta* fourth stage larvae (*Tci*-L4ES). Furthermore, we perform a comparative proteomic analysis of vesicle-enriched and vesicle-free *Tci*-L4ES. Approximately 73% of the proteins identified in the vesicle-enriched fraction were unique to this fraction, whilst the remaining 27% were present in both vesicle-enriched and vesicle-free fraction. These unique proteins included structural proteins, nuclear proteins, metabolic proteins, proteolytic enzymes and activation-associated secreted proteins. Finally, we demonstrate that molecules present within the vesicles-enriched material are targets of the IgA and IgG response in *T. circumcincta* infected sheep, and could potentially represent useful targets for future vaccine intervention studies.

## Highlights

• Presence of microvesicles in *T. circumcinctas* L4 excretory/secretory (ES) products.
• 73% of the microvesicle proteins were not identified in previous ES studies.
• Several proteins were identified with potential immunomodulatory function.
• Some of the proteins identified, were targets of the host immune response.

## Graphical abstract

**Figure:**
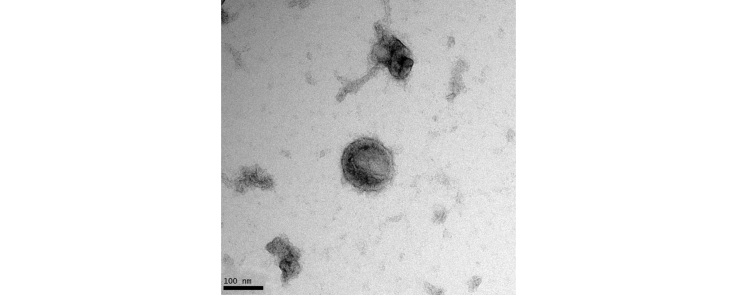

## 1 Introduction

*Teladorsagia circumcincta* is the principal cause of ovine parasitic gastroenteritis (teladorsagiosis) in temperate climatic regions and has been reported as the most predominant nematode species in ovine flocks in the UK (Bartley et al., 2003, Burgess et al., 2012). In the northern hemisphere, teladorsagiosis is usually seen from July to October, coincident with an exponential increase in infective third stage larvae (L3) on the pasture on which the animals graze. The main clinical manifestations are reduced weight gain/condition loss and dehydration due to diarrhoea (Scott, 2007). Faecal contamination of the fleece in the perineal area may also attract blowflies, which can lead to myiasis. Teladorsagiosis has a significant economic impact on the industry: studies have estimated that losses in excess of £84 million *per annum* in the UK are associated with ovine parasitic gastroenteritis due to reduced productivity and the cost of the treatment alone (Nieuwhof and Bishop, 2005), with *T. circumcincta* being the major contributor. Moreover, the cost of sub-clinical infection, which is likely to be significant, is not included in the above figures (Nieuwhof and Bishop, 2005).

Control of *T. circumcincta* is largely based on the administration of broad spectrum anthelmintics (Kohler, 2001); however, resistance to these drugs appears to be widespread including reports of multiple drug resistant *T. circumcincta* isolates (Sargison, 2011, Wrigley et al., 2006). The introduction of two new classes of anthelmintic, monepantel in 2008 (Kaminsky et al., 2008) and derquantel in 2010 (Little et al., 2010), have helped to fill the gaps created in the control management of parasitic helminths. However, recent studies have shown that *T. circumcincta* and *Trichostrongylus colubriformis* have developed resistance to monepantel (Scott et al., 2013) and although derquantel is still effective in sheep, experience from other anthelmintic drug classes would suggest that development of resistance to derquantel is likely.

Vaccination represents an alternative control strategy against *T. circumcincta* as sheep develop protective immunity against the parasite after experimental multiple infections (Seaton et al., 1989, Smith et al., 1983). Recent work has identified several vaccine candidates (Nisbet et al., 2010a, Nisbet et al., 2009, Nisbet et al., 2010b, Nisbet et al., 2011, Redmond et al., 2006) and a “cocktail” of eight recombinant proteins targeting the fourth larval stage of *T. circumcincta*, which is the stage most intimately associated with the host, has been shown to stimulate significant levels of protection against experimental *T. circumcincta* challenge (Nisbet et al., 2013). However, the levels of protection achieved using this recombinant protein vaccine were variable, with mean reductions in egg output and adult worm burdens ranging from 58 to 70% and 56 to 75%, respectively, over two replicated experimental trials (Nisbet et al., 2013). These data indicate that further optimisation of the vaccine may be required.

Extracellular vesicles (EVs) are membrane vesicles which originate from either endosomal membranes (exosomes) or plasma membranes (microvesicles) (Raposo and Stoorvogel, 2013). These vesicles exhibit differences size ranges, with exosomes ranging between 30 and 100 nm (Thery et al., 2006, Urbanelli et al., 2013) and microvesicles ranging between 100 and 1000 nm in size (Muralidharan-Chari et al., 2010). EVs can be secreted by multiple mammalian cell types and the nature and function of these vesicles, and exosomes in particular, has been intensively studied over the last decade (Bobrie et al., 2011, Couzin, 2005, Mathivanan et al., 2010, Montaner et al., 2014, Simons and Raposo, 2009, Thery, 2011). Growth in this field is largely due to the discovery that exosomes play a key role in intercellular signalling and cell–cell communication; for example, B-lymphocytes (Raposo et al., 1996) and dendritic cells (Zitvogel et al., 1998) secrete exosomes which contain molecules that affect host immune responses (Andreola et al., 2002, Thery et al., 2002).

Research regarding EVs has now expanded to other organisms including parasitic helminths (Montaner et al., 2014). Studies showed that the trematodes *Fasciola hepatica* and *Echinostoma caproni* actively release exosomes (Chaiyadet et al., 2015, Marcilla et al., 2012), as do the nematode species, *Caenorhabditis elegans* (Liegeois et al., 2006), *Heligmosomoides polygyrus* (Buck et al., 2014) and *Trichuris suis* (Hansen et al., 2015). Marcilla et al. (2012) showed that rat intestinal cells actively take up exosomes secreted by *F. hepatica* and *E. caproni*. A recent study demonstrated that small RNAs contained in exosomes secreted by *H. polygyrus* can regulate genes of the host (mouse) innate immune system (Buck et al., 2014). These observations support the hypothesis that exosomes play roles in host-parasite communication. Furthermore, based on these observations, it has been suggested that exosomes could contain candidates for vaccines and/or targets for pharmaceutical intervention (Marcilla et al., 2012). To our knowledge there have been no reports on whether ruminant parasitic nematodes release EVs.

The work here examined whether EVs could be found in the excretory/secretory (ES) products of *T. circumcincta*. We focused on ES material from fourth stage larvae (L4) as previous studies have indicated that local humoral and cellular responses to this larval stage are critical in the acquisition of protective immunity in lambs (Smith et al., 1986, Smith et al., 1985, Stear et al., 1995, Strain et al., 2002). Furthermore, a detailed proteomics analysis was conducted to characterize the contents of these vesicles. Finally, an important consideration was to examine whether the vesicle proteins are targets of the host immune response, which would provide evidence of their release *in vivo*. Thus, immunoblots were performed to examine the immunogenicity of the contents of EVs released by *T. circumcincta*.

## 2 Materials and methods

### 2.1 Parasite material

EVs were prepared from the ES products of *T. circumcincta* L4 (*Tci*-L4ES). The L4 were harvested following methods described previously (Knox and Jones, 1990, Redmond et al., 2006, Smith et al., 2009). Briefly, 5 helminth-free lambs were infected, each with approximately 150,000 *T. circumcincta* L3 (strain MTci2; an anthelmintic-susceptible laboratory isolate from Moredun Research Institute). Seven days later, the lambs were euthanized to retrieve the L4. Each abomasum was removed and processed individually. The L4 were retrieved from the mucosa and from the abomasal contents as described previously (Knox and Jones, 1990). The L4 were pooled, washed with Phosphate Buffered Saline (PBS) and cultured in nematode culture medium [RPMI 1640 Gibco^®^, Life technologies™ (500 ml) supplemented with sterile l- glutamine (10 ml of 100 mM), D-glucose solution (5 g in 50 ml), penicillin/streptomycin (5 ml of 10,000 μg/ml), amphotericin B (62.5 mg), gentamycin sulphate (12.5 mg) and Hepes solution (10 ml; 1 M Sigma-Aldrich)] as described in previously published methods (Redmond et al., 2006).

The protocol for the collection of the ES material after 24, 48 and 72 h of culture was based on previously published studies (Smith et al., 2009). The pooled ES products from these time-points were filtered using a 0.22 μm syringe filter (millex^®^ GP). The filtered ES products were divided into two equal aliquots (one to be processed for EVs purification and the other for total *Tci*-L4ES preparation) and frozen at −80 °C until further processing.

### 2.2 Extracellular vesicle purification

EVs purification was carried out following the protocol described in Buck et al. (2014). Briefly, *Tci*-L4ES samples were thawed at room temperature and ultracentrifuged at 100,000 × *g* for 2 h using a SW40 swing out rotor (cooled overnight at 4 °C). The supernatant (termed EV-free *Tci*-L4ES) was removed and stored at −80 °C prior to processing. Pelleted material was washed twice with 12.5 ml PBS. After each wash, the samples were ultracentrifuged at 100,000 × *g* for 2 h and the supernatant was removed. Finally, pelleted material was re-suspended in 100 μl PBS (termed EV-enriched *Tci*-L4ES) and protein concentration measured with Qubit^®^ 2.0 Fluorometer (Life technologies™). An aliquot of the pelleted material was immediately processed for microscopy as described below, and the remaining material stored at −80 °C prior to proteomic analysis.

### 2.3 Transmission electron microscopy (TEM)

An aliquot (8 μl) of the pelleted material from 2.2 was fixed with an equal volume of paraformaldehyde 4% (Fisher scientific). The sample was prepared by the Electron Microscopy (EM) unit of the University of Edinburgh (School of Biological Sciences, King’s Buildings). The technicians of the EM unit prepared the sample for the TEM as described in Buck et al., 2014.

### 2.4 Concentration of total and EV-free Tci-L4ES

Total *Tci*-L4ES and EV-free *Tci*-L4ES were thawed at room temperature and Amicon Ultra-15 centrifugal filter units (MWCO 10 kDa; Sigma Aldrich) were used to concentrate the material according to the manufacturer’s instructions to a final volume of ∼1 ml. Finally, the filter units were washed twice with pre-chilled PBS to buffer exchange the material into PBS. The concentration of the protein in these samples was measured using the Pierce^®^ BCA Protein Assay kit (Thermo scientific) based on the manufacturer’s protocol.

### 2.5 Protein profiles of the EV-enriched, the EV-free and total Tci-L4ES products

To examine the protein profile of EV-enriched, EV-free and total *Tci*-L4ES, 2 μg of each sample were subjected to electrophoresis. The samples were denatured at 70 °C for 10 min after adding NuPAGE^®^ LDS sample buffer (4X; Life Technologies™) and 10X NuPAGE^®^ sample reducing agent (Life Technologies™). Following the denaturing step, the samples (2 μg protein; 10 μl total volume each sample) were subjected to electrophoresis on NuPAGE^®^ Novex^®^ Bis-Tris 4–12% mini gels (Life Technologies™) under reducing conditions using NuPAGE^®^ MES SDS running buffer (Life Technologies™). The proteins were stained with SimplyBlue™ SafeStain (Life Technologies™) based on the manufacturer’s instructions. An identical replicate NuPAGE^®^ gel was prepared as above and the proteins were stained with SilverQuest™ Silver Staining Kit (Life Technologies™) according to the manufacturer’s instructions.

### 2.6 Proteomic analysis of EV-enriched and EV-free Tci-L4ES

Preparations that were confirmed to contain vesicles by TEM (EV-enriched *Tci*-L4ES) and concentrated EV-free *Tci*-L4ES were subjected to proteomic analysis. Approximately 10 μg of the EV-enriched and 10 μg of the EV-free *Tci*-L4ES products were prepared, subjected to electrophoresis on NuPAGE^®^ Novex^®^ Bis-Tris 4–12% mini gels (Life Technologies™) and stained with SimplyBlue™ SafeStain (Life Technologies™) as described above (section 2.5). The intact NuPAGE^®^ gel was submitted to the Moredun Proteomics Facility (Moredun Research Institute) for liquid chromatography-electrospray-ionisation tandem mass spectrometry (LC-ESI–MS/MS) analysis as described previously (Smith et al., 2009). Deconvoluted MS/MS data in.mgf (Mascot Generic Format) format was imported into ProteinScape™ V3.1 (Bruker Daltonics) proteomics data analysis software for downstream database mining of both Nembase4 (http://www.nematodes.org/nembase4/) and a cognate *T. circumcincta* transcriptomic database (Ellis et al., 2014) utilising the Mascot™ V2.4.1 (Matrix Science) search algorithm. The protein content of each individual gel slice was established using the “Protein Search” feature of ProteinScape™, whilst separate compilations of the proteins contained in all 24 gel slices for each sample were produced using the “Protein Extractor” feature of the software. Mascot search parameters were set in accordance with published guidelines (Taylor and Goodlett, 2005) and to this end, fixed (carbamidomethyl “C”) and variable (oxidation “M” and deamidation “N,Q”) modifications were selected along with peptide (MS) and secondary fragmentation (MS/MS) tolerance values of 0.5 Da whilst allowing for a single 13C isotope. Molecular weight search (MOWSE) scores attained for individual protein identifications were inspected manually and considered significant only if a) two peptides were matched for each protein, and b) each matched peptide contained an unbroken “*b*” or “*y*” ion series represented by of a minimum of four contiguous amino acid residues. Overall confidence in protein identifications was enhanced by re-searching the MS data against a decoy database constructed through the False Discovery Rate (FDR) feature of the Mascot search algorithm. Setting the FDR at a stringency of 1% did not result in the exclusion of any proteins that were confidently identified in the original database search and validation. The results in ProteinScape™ from the Nembase4 search included the sequence ID of each protein. Subsequently, each protein sequence from Nembase4 was subjected to a manual Blast procedure (tblastn) against the full NCBInr database to find the closest homologous protein. Finally, the proteins detected in the EV-enriched sample were searched in the ExoCarta database (http://www.exocarta.org/; Mathivanan and Simpson, 2009, Mathivanan et al., 2012), and in previous publications (Marcilla et al., 2012) to examine whether they were detected previously as exosome-related proteins. Moreover, they were searched against the results of previous studies that examined *T. circumcincta* L4-specific gene expression (Nisbet et al., 2008) and the protein profile of total *Tci*-L4ES (Smith et al., 2009).

### 2.7 Immunogenicity of the EV-enriched fraction

Immunoblots were performed to test whether serum IgG and IgA from sheep which had been trickle infected with 2000 *T. circumcincta* L3 three times per week for 4 weeks (Nisbet et al., 2013), bound components of the EV-enriched fraction. Serum samples were collected at 6 weeks post-infection at a time where immunity to *T. circumcincta*, as determined by decreasing faecal parasite egg output, had developed. Sera from the same sheep before infection (‘pre-immune’) were used as a negative control. The immunoblots were conducted as described by Nisbet et al. (2010b). Briefly, 5 μg of each sample (EV-enriched, EV-free and total *Tci*-L4ES products) were prepared and subjected to electrophoresis under reducing conditions as described above (section 2.5). The proteins were transferred onto nitrocellulose membranes (0.2 μm pore size; Life Technologies™); the membranes washed briefly in TNTT (10 mM Tris, 0.5 M NaCl, 0·05% Tween 20, 0.01% thiomersal, pH 7.4) and incubated in TNTT overnight at 4 °C. The blots were incubated with sheep sera, diluted 1:200 in TNTT, for 1 h before being washed three times in TNTT (10 min/wash); incubated for 1 h in donkey anti-sheep/goat IgG horseradish peroxidase (HRP) conjugate (AbD Serotec) diluted 1:1000 in TNTT; and washed three times in TNTT (10 min/wash). Peroxidase activity was revealed using 3,3′-Diaminobenzidine (DAB) as substrate. Essentially the same protocol was used for the IgA immunoblot but the secondary antibody was mouse anti-bovine/ovine IgA (clone K84.2F9, AbD Serotec) diluted 1:1000 in TNTT followed by a tertiary antibody [polyclonal rabbit anti-mouse immunoglobulins/HRP conjugate (Dako) diluted 1:1000 in TNTT]. Peroxidase activity was revealed using the Amersham ECL Western Blotting system (GE healthcare Life sciences) following the manufacturer’s protocol.

## 3 Results

### 3.1 Identification of extracellular vesicles in Tci-L4ES

**Fig. 1:**
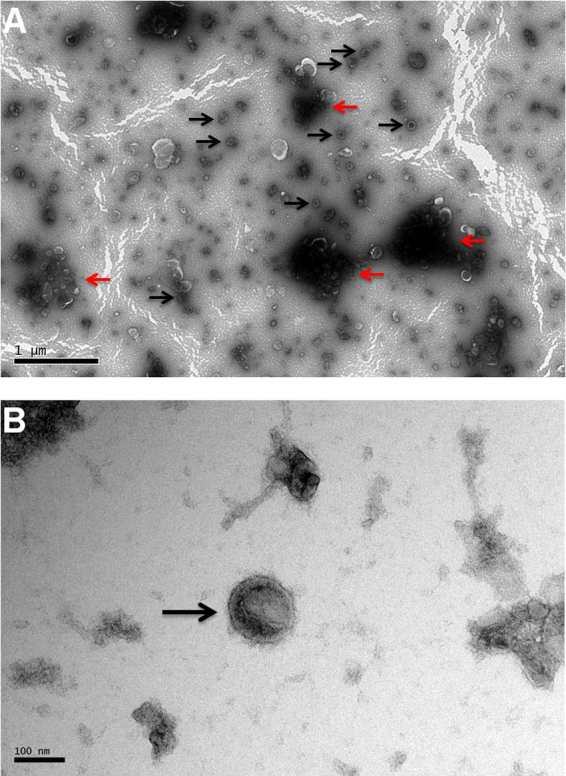
Extracellular vesicles in the ES products of *T. circumcincta* L4 stage. Panel A shows vesicles spread across the optical field (examples indicated with black arrows). Vesicles tend to aggregate which can be seen as the darker areas in panel A (examples indicated with red arrows). Panel B shows one vesicle in higher magnification (black arrow). (For interpretation of the references to colour in this figure legend, the reader is referred to the web version of this article.)

**Fig. 2:**
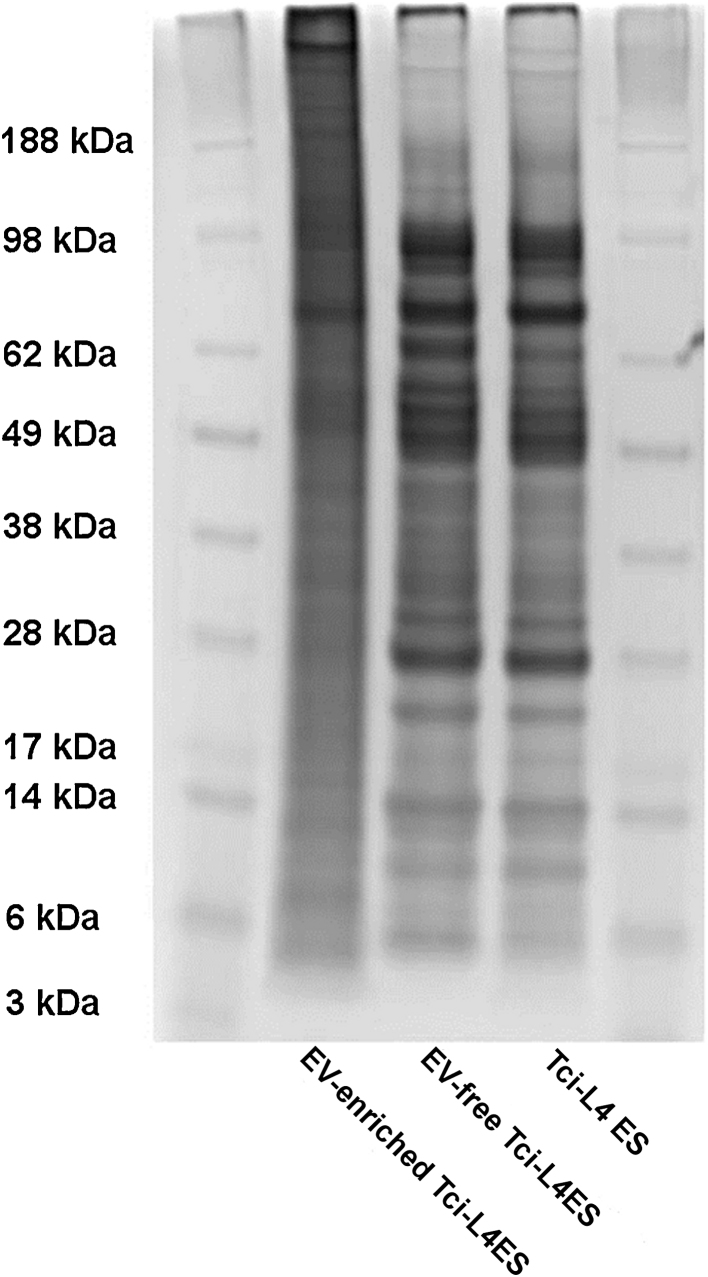
Protein profiles of EV-enriched, EV-free and total *Tci*-L4ES products of *T. circumcincta* L4 stage. 2 μg of each sample were subjected to electrophoresis under reducing conditions. Subsequently, the proteins were stained with SilverQuest™ Silver Staining Kit (Life Technologies™).

The TEM confirmed the presence of vesicles in *Tci*-L4ES collected from sheep at 1 week post infection. The majority of the vesicles were visible as small membrane vesicles within the size range (30–100 nm) of exosomes (Urbanelli et al., 2013; arrowed, Fig. 1, panels A and B). One-dimensional protein profiles obtained for the EV-enriched, EV-free and total *Tci*-L4ES (Fig. 2) showed that the profile for the EV-enriched fraction was, qualitatively, substantially different from those of EV-free and total ES preparations.

### 3.2 Proteomic analysis of the EV-enriched and EV-free Tci-L4ES

**Fig. 3:**
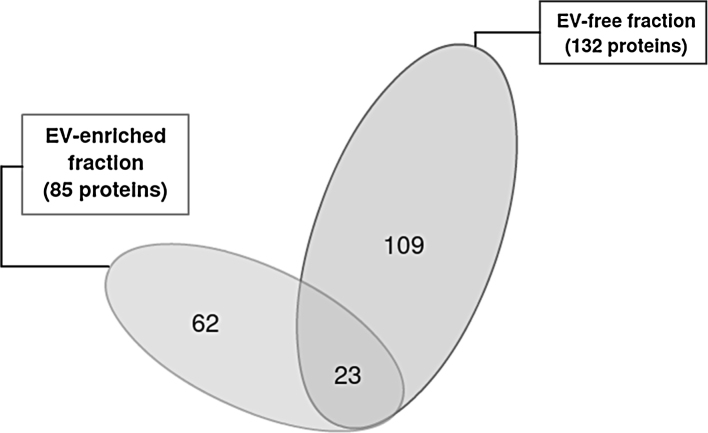
Venn diagram summarizing the number of unique and common proteins in the EV-enriched and EV-free fractions.

**Fig. 4:**
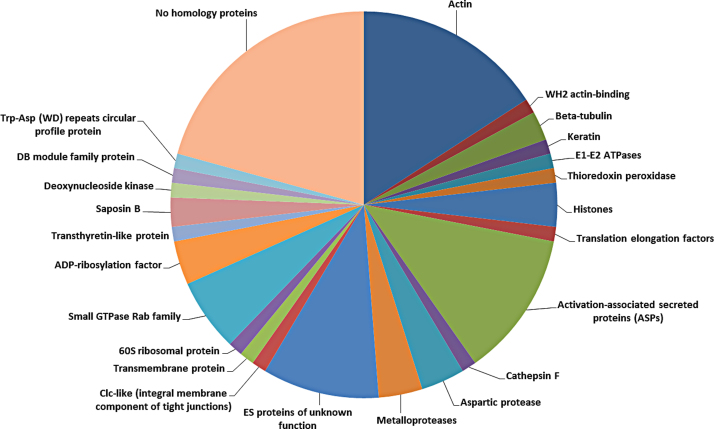
Summary of the protein groups identified in the EV-enriched *Tci*-L4ES fraction.

Manual inspection of the proteomics dataset revealed 85 and 132 confidently identified proteins in the EV-enriched and EV-free fractions, respectively. A total of 23 proteins were common to the two fractions and the remainder were unique to each (Fig. 3). As the aim here was to characterize EVs released by *T. circumcincta* L4, the subsequent analysis focussed on proteins identified in the EV-enriched sample. The proteins identified in the EV-enriched and the EV-free fractions and their closest homologues are available in supplementary files S1 and S2, respectively. The proteins of the EV-enriched fraction were grouped according to broad function and these groups are shown in Table 1 and Fig. 4. The protein groups were as follows: structural proteins (20.7% of the proteins); metabolic proteins (2.4%); nuclear proteins (4.9%); activation-associated secreted proteins (ASPs; 12.2%); proteolytic enzymes (8.6%); ES proteins of unknown function (9.8%); cell-to-cell, cell-to-matrix interaction proteins (2.4%); ribosomal proteins (1.2%); Rab GTPases (6.1%); proteins for which homologues could be identified but could not be classified in the previous categories (11%); and proteins for which no homologues could be identified (20.7%).

### 3.3 Immunogenicity of EV-enriched, EV-free and total Tci-L4ES

**Fig. 5:**
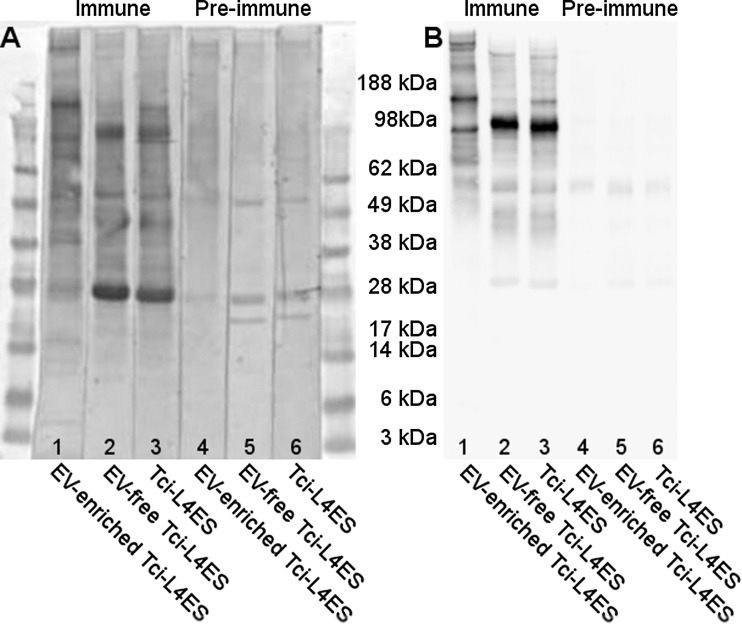
Immunoblots of EV-enriched, EV-free and total *Tci*-L4ES products of *T. circumcincta* L4 stage probed with sera obtained from *T. circumcincta* trickle infected sheep. Panel A: IgG immunoreactivity; Panel B IgA immunoreactivity. Both panels − Lanes 1–3, probed with sera from trickle-infected sheep; lanes 4–6 probed with sera taken prior to the trickle infections.

Immunoblotting experiments demonstrated that proteins present in the EV-enriched fraction, EV-free and total *Tci*-L4ES were bound by IgG (Fig. 5; panel A) and IgA (Fig. 5; panel B) present in sera from *T. circumcincta* infected sheep, but not in sera from these sheep prior to infection. In both immunoblots, there were bands that were strongly bound by IgG or IgA either in the EV-enriched and the total *Tci-*L4ES only (e.g. area between 62 and 98 kDa) or only in the EV-free and total *Tci-*L4ES (e.g. area between 98 and 188 kDa or 28 kDa area). The immunoblot results suggest that the higher molecular weight molecules are bound more strongly by serum IgA, whilst a wider range of molecular weight molecules are bound by serum IgG.

## 4 Discussion

The work here demonstrated that EVs are present in the ES products from *T. circumcincta* L4. This developmental stage was selected, as it is intimately associated with the abomasal glands of the sheep host (Denham, 1969). Moreover, previous studies suggest that L4 are targeted by local humoral and cellular immune responses that are critical in the development of protective immunity (Smith et al., 1986, Smith et al., 1985, Stear et al., 1995, Strain et al., 2002). Shotgun proteomics analysis revealed that approximately 70% of the identified proteins detected in the EV- enriched fraction were potentially unique to these components with no detectable presence in the EV-free ES products. Conversely, the remaining 30% of the proteins identified in the EV-enriched fraction were also identified in EV-free ES. Finally, some contents of the EVs were found to be immunogenic, as they were shown to be bound by IgG and IgA present in sera obtained from *T. circumcincta* infected sheep.

A proportion of the vesicles identified in *Tci*-L4ES exhibited the morphological characteristics of exosomes with a size that varied from 30 to 100 nm (Urbanelli et al., 2013). Although exosomes have been reported in other parasitic helminths (Bernal et al., 2014, Buck et al., 2014, Chaiyadet et al., 2015, Hansen et al., 2015, Marcilla et al., 2012, Wang et al., 2015) this is the first report, to our knowledge, of extracellular vesicle production and release by a nematode that infects ruminants. Following TEM confirmation of EVs being present in the ES products, the protein profile of the EV-enriched sample was compared with protein profiles of EV-free and total *Tci*-L4ES products. The results suggested that the protein profile of the EV-enriched sample was different to the profiles of the other two samples, and that the former sample contained protein bands that were not identified in the EV-free *Tci*-L4ES by gel electrophoresis and staining.

The protocol that we followed did not include an initial lower speed centrifugation to separate the larger vesicles. Alternatively, we used a 0.22 μm filter. The aggregations mentioned (Fig. 1) might reflect a mixture of microvesicle fragments and exosomes due to the break up of larger vesicles with the filter. As a result, we might have missed larger vesicles and the EV populations might have been different if an initial lower speed centrifugation was performed. Future EV studies should follow protocols that allow purification of different vesicle populations and could include more accurate methods of assessing EV size and total concentration, such as tunable resistive pulse sensing (Anderson et al., 2015) or nanoparticle tracking analysis (Dragovic et al., 2011). The EV recovery in our study was measured based on the protein content and this might be considered as problematic since we re-suspended the EVs in PBS which does not remove potential contaminating protein complexes, proteins or lipoproteins. Nevertheless, the proteins that we detected in the EV-enriched and EV-free fractions were considerably different which gives us confidence that the proteins identified within the EV-enriched fraction were of EV origin rather than from contaminating proteins within the ES supernatant.

The proteomic analysis of the EVs revealed a total of 85 proteins in the EV-enriched sample. Of these, 23 proteins were also found in the EV-free sample. It is not yet clear whether this set of 23 proteins which were common to both preparations were adventitious or adherent to the EVs during purification or if they represent a set of proteins that are released both in solution in the ES and in the EVs. The 82 proteins identified in the EV-enriched fraction included, among others, structural proteins (e.g. actin, β-tubulin); metabolic proteins (e.g. peroxiredoxin); nuclear proteins (e.g. histone); ASPs; Rab GTPases; metallopeptidases; and several proteins for which no homologues could be identified. Genes encoding 56% of the characterised vesicle proteins were identified in a previous transcriptomic study performed on *T. circumcincta* L4 harvested at 8 days post-infection, but not in the L3 stage (Nisbet et al., 2008). Some of the remaining vesicle proteins have not been identified previously in *T. circumcincta* and only 16% of the vesicle proteins characterised here were identified in a proteomic analysis of total ES products derived from *T. circumcincta* larvae harvested at 1-, 3- and 5 days post infection (Smith et al., 2009).

In terms of comparison with EVs derived from other organisms, the analysis here showed that 76% of the EV proteins identified in the current study were among molecules previously identified in the ExoCarta database (Marcilla et al., 2012, Mathivanan et al., 2012, Mathivanan et al., 2010, Simpson et al., 2012) and in other published studies relating to exosomes in helminths (Bernal et al., 2014, Marcilla et al., 2012). The remaining 24% included proteins such as ASPs and ES proteins of unknown function (i.e. those previously identified in ES material from other parasite species).

Actins and beta-tubulin are structural proteins which represented 16% and 2% of the proteins identified in the EV-enriched sample, respectively, and have been identified in vesicles of other organisms (Mathivanan et al., 2012, Mathivanan and Simpson, 2009). Actins are associated with the cytoskeletal microfilaments and beta-tubulin with the cytoskeletal microtubules (Schappi et al., 2014). Keratin, another cytoskeletal protein, has been also identified in exosomes originating from human saliva (Xiao and Wong, 2012) and represented 1% of the proteins identified. The identification of keratin in the proteomic analysis could indicate human contamination, however, the top hit after a tBLASTn search of the protein identified in the EV-enriched sample against NCBInr was an *Ostertagia ostertagi* partial mRNA for keratin (AJ429146.1, 63% identities, E value = 2e-71). A subsequent tBLASTn search of the protein identified in the exosome-enriched sample against the human nucleotide collection resulted in no significant similarities. WH2 actin-binding protein (1% of the proteins identified here), associated with actin assembly, has not been detected in exosomes from any other organism based on information in ExoCarta (Carlier et al., 2013). Nevertheless, there are other actin-binding proteins in the ExoCarta database, such as coronin and moesin. It has been suggested that these structural proteins might be associated with the exosome production (Wubbolts et al., 2003).

The metabolic proteins that were identified in the *T. circumcincta* EV-enriched fraction (Na/K-ATPase and thioredoxin peroxidase; 1% of the proteins each) are also present in the ExoCarta database. Ce-EAT-6 is a Na/K-ATPase and has been shown to interact with ATP and to have sodium:potassium-exchanging ATPase activity (Davis et al., 1995). *C. elegans Ce-eat-6* mutations have been associated with paralysis of the pharyngeal muscles in *C. elegans* (Davis et al., 1995). Moreover, studies in the trematode, *Schistosoma mansoni*, have shown that Na/K-ATPase has been associated with the acquisition of resistance to complement after the parasite has penetrated the skin (Tarrab-Hazdai et al., 1997) but there are no published studies to show whether or not this ATPase is found in the EVs of this species.

Thioredoxin peroxidase, also known as peroxiredoxin, was identified in the *T. circumcincta* EV-enriched sample. This is an antioxidant enzyme that is considered as a damage-associated molecular pattern molecule (DAMP), but also as a pathogen-associated molecular pattern molecule (PAMP) (Medzhitov, 2007, Robinson et al., 2010b). Peroxiredoxins are thought to play a role in the survival of parasites against reactive oxygen species generated by the immune system of the host (Gretes et al., 2012). It has also been proposed that helminth peroxiredoxins are homologous to host DAMPs and are responsible for the modulation of the immune response (Dalton et al., 2013, Robinson et al., 2010b). In *F. hepatica*, these molecules have been shown to help direct the immune system towards a Th2-type response, thought to be favourable for the parasites’ development in the host (Dalton et al., 2013, Robinson et al., 2010a). Peroxiredoxin has also been found in exosomes of *F. hepatica* and in *E. caproni* (Marcilla et al., 2012). Peroxiredoxin has been proposed as a potential vaccine candidate in *Fasciola* spp., due to its immunomodulatory properties (Robinson et al., 2010a, Robinson et al., 2010b) but vaccine trials with recombinant peroxiredoxin in goats and buffaloes did not show significant levels of protection (Mendes et al., 2010, Raina et al., 2011).

ASPs represented a notable proportion (12%) of the proteins identified in the EVs. These proteins have not yet been described in the ExoCarta database, which could be explained by the fact that ASPs are nematode-specific (Parkinson et al., 2004). The precise function of nematode ASPs is still unknown but it is suggested that these proteins might act as virulence factors that manipulate host immune responses and contribute to parasite survival in the host (Asojo et al., 2005, Hawdon et al., 1996, Hawdon et al., 1999). Moreover, ASPs have been identified as vaccine candidates against several parasitic nematodes, including *T. circumcincta* (Geldhof et al., 2002, Ghosh et al., 1996, Kooyman et al., 2000, Nisbet et al., 2013). Here, 10 ASPs were identified in the exosome-enriched sample (*tdc00434*, *tdc00460*, *tdc00472*, *tdc00533*, *tdc00656*, *tdc00942, tdc02274*, *tdc02406*, *tdc02610* and *tdc02887*). These proteins were not the same as the ASPs identified by immunoscreening of L4 *T. circumcincta* ES harvested at 5 days post-infection (Nisbet et al., 2010b). This might have been due to the low concentration of exosomes in total ES products and/or their relatively poor immunogenicity. As a result, the novel ASPs revealed from the current proteomic analysis might be potential vaccine targets.

Another group of proteins, that represented 10% of the proteins identified in the EV-enriched material and have not been previously identified in exosomes of other organisms, are ‘nematode ES proteins’ ascribed no specific function on the basis of the absence of characteristic motifs in their sequences. However, TDP01869 was found to share homology with the *H. contortus* 15 kDa ES protein (Schallig et al., 1997b); HCP02856, HCP06214 and TDP00176 homologues of the *T. circumcincta* 20 kDa ES protein (Nisbet et al., 2013); OOP00884, TDP00436 and TDP00589 homologues of the *O. ostertagi* ‘putative L3 ES protein’ (De Maere et al., 2002); and TDP00713 homologue of the *T. colubriformis* 30 kDa glycoprotein (Savin et al., 1990). Each of the homologous proteins (*H. contortus* 15 kDa ES protein, *T. circumcincta* 20 kDa ES protein, *O. ostertagi* ‘putative L3 ES protein’ and *T. colubriformis* 30 kDa glycoprotein) has been described as a vaccine candidate in previous studies. In particular, in an immunisation trial in sheep using the two native *H. contortus* ES proteins (the 15 kDa and 24 kDa ES proteins), significant protection against challenge was observed in vaccinated sheep when compared with challenge control animals (Schallig et al., 1997a). The *T. colubriformis* 30 kDa glycoprotein was found to be protective in guinea pigs against challenge and was found to reduce worm burdens by 59% in vaccinates compared to challenge controls (Savin et al., 1990). Furthermore, the *T. circumcincta* 20 kDa ES protein (Tci-ES20) is one of the components of the eight-protein recombinant ‘cocktail’ mentioned above (Nisbet et al., 2013). The *O. ostertagi* ‘putative L3 ES protein’ has been proposed as potential protective antigen because it was bound by antibodies from “immune” calves (De Maere et al., 2002), but it remains to be tested in an immunisation trial. Putative functions for the above proteins remain to be established; however, their involvement in several successful vaccine trials indicates their potential as valid candidates.

Cathepsin F (Tci-CF-1) was found here in the EVs of *T. circumcincta* (1%). Tci-CF-1 is the most abundant molecule to be identified in the ES products of *T. circumcincta* larvae (including those harvested at 5, 6 and 9 days post infection) and has been found to be a target of IgA responses in previously infected sheep (Redmond et al., 2006). A related protein has been identified in the exosomes of *F. hepatica* (Cathepsin L or FheCL; Marcilla et al., 2012). FheCL has been associated with tissue penetration during the larvae migration through the host tissue (Dalton and Heffernan, 1989), nutrition (Dalton and Heffernan, 1989, Smith et al., 1993b) and/or immunomodulation (Chapman and Mitchell, 1982, Dalton and Heffernan, 1989, Smith et al., 1993a). A more recent study has shown that FheCL suppressed Th1 immune responses by reducing the production of IFN-γ (O'Neill et al., 2001). It has been suggested that Tci-CF-1 may have a similar immunomodulatory function (Redmond et al., 2006). Tci-CF-1 and FheCL have been tested in trials, which showed significant levels of protection against *T. circumcincta* in lambs, as part of the eight-protein “cocktail”, (Nisbet et al., 2013) and *F. hepatica* in calves (Dalton et al., 1996), respectively.

Rab GTPases represented 6% of the proteins found in the EVs analysed here. These proteins have been highlighted in previous studies in helminths (Buck et al., 2014) and were identified in other organisms in the ExoCarta database search. In 2010, there were 40 Rab proteins in the ExoCarta database from various studies on exosomes (Mathivanan et al., 2010). Similar numbers are present in the database currently. Rab proteins are the largest family of small GTPases and have been associated with the exosome secretion pathway in cells, for example Rab27 in Hela cells (Ostrowski et al., 2010), Rab35 in oligodendroglial cells (Hsu et al., 2010) and Rab11 in erythroleukemia cell lines (Savina et al., 2005). Different Rab proteins are involved with different stages of the exosome secretion pathway (Stenmark, 2009). For example, Rab4, Rab5 and Rab11 are involved in the early stages, whilst Rab7 and Rab9 in the later stages (Stenmark, 2009).

The group of proteins with no homology hits identified comprised the largest proportion of proteins in the EV-enriched fraction (21%). These proteins did not have any motifs identified in their sequence that would indicate a potential function. A previous study has also identified proteins with no homology and unknown function (17% of the vesicle proteins) in *D. dendriticum*- derived exosomes (Bernal et al., 2014).

An important finding here was the demonstrable immunogenicity of components of the EV-enriched *Tci*-L4ES following their incubation with serum harvested from sheep trickle-infected with *T.circumcincta* over a 6week period. Immunoblotting revealed that vesicle components were bound by IgG and IgA. This is an indication that the EVs are secreted *in vivo* and are not an artefact from *in vitro* culture of the parasites. To our knowledge, this is the first demonstration in any parasite species that exosome components are targeted by the host antibody response. Thus, it can be suggested that some of the molecules in the EV-enriched samples could be vaccine targets since they are bound by the immune system of the host during the development of a protective response. Nevertheless caution is needed because, the immune-recognised proteins of the EV-enriched *Tci*-L4ES were not identified and the results could be due to the expression of those proteins in worm locations other than EVs which are exposed to the host immune response.

## 5 Conclusion

EVs are released by *T. circumcincta* L4. The proteomic analysis revealed several proteins with potential immunomodulatory function, supporting the earlier hypothesis that helminths use EVs to simultaneously deliver multiple antigens into host cells and regulate their function (Dalton et al., 2013). Finally, the binding of proteins within the EV-enriched fractions by IgA and IgG of previously infected sheep confirmed their immunogenicity.

## Conflict of interest

The authors declare that there is no conflict of interest.

## Figures and Tables

**Table 1:** Broad Functional grouping of proteins identified in the EV-enriched *Tci*-L4ES.

Protein	Number of proteins
1. Structural proteins	
Actin^a^, ^c^	13
WH2 actin-binding	1
Beta-tubulin^c^	2
Keratin^a^, ^c^	1
2. Metabolic Proteins	
E1-E2 ATPases^c^	1
Thioredoxin peroxidase^a^, ^c^	1
3. Nuclear proteins	
Histones^c^	3
Translation elongation factors^c^	1
4. Activation-associated secreted proteins	
ASPs^a^, ^b^	10
5. Proteolytic enzymes	
Cathepsin F^a^, ^b^, ^c^	1
Aspartic protease^c^	3
Metalloproteases^a^, ^b^, ^c^	3
6. Excretory/Secretory (ES) proteins of unknown function	
ES proteins of unknown function^a^	8
7. Cell to cell, Cell to matrix interactions	
Clc-like (integral membrane component of tight junctions)^a^, ^c^	1
Transmembrane protein^a^, ^c^	1
8. Ribosomal proteins	
60S ribosomal protein^a^, ^c^	1
9. Rab GTPases	
Small GTPase Rab family^c^	5
10. Other function	
ADP-ribosylation factor^c^	3
Transthyretin-like protein^a^, ^c^	1
Saposin B^a^	2
Deoxynucleoside kinase	1
DB module family protein	1
Trp-Asp (WD) repeats circular profile protein^c^	1
11. No homology	
No homology proteins^a^, ^b^, ^c^	17

a Indicates the identification of genes belonging to the same groups by Nisbet et al., 2008.

b Indicates the identification of genes belonging to the same groups by Smith et al., 2009.

c Indicates the identification of the proteins in exosomes derived by other organisms (ExoCarta database and Marcilla et al., 2012).
